# Evaluation and Improving the Quality of Arterial Blood Gas Interpretation Among Junior Doctors in Aswan University Hospital: A Clinical Audit

**DOI:** 10.7759/cureus.74906

**Published:** 2024-12-01

**Authors:** Eiman Yassir Musa Hussain, Ahmed Mahmoud Sidahmed Abdullah, Razan Mutasim Mahgoub Idris, Ziyad Tarig Hashim Gabir, Reem Ahmed Mohammed Diab, Rana Noureldaim Mustafa Ahmed, Khalid Abdelhadi Ahmed Elbalal, Othman Yousuf Ibrahim Elhaj, Mohammedalmojtaba Hamza Mohammed Abdelmagid, Rashed Abdulhafith Mahmoud Ahmed, Ammar Yasir Mohamed Abdelhai, Randa Mahamoud Hamid Hassan, Shazly Baghdadi Ali Ahmed

**Affiliations:** 1 Pulmonary Medicine, Aswan University Hospitals, Aswan, EGY

**Keywords:** acid-base balance, arterial blood gas (abg), clinical audit, diagnostic accuracy, healthcare education

## Abstract

Background: Arterial blood gas (ABG) analysis is a vital diagnostic tool in clinical settings, particularly for assessing oxygenation, ventilation, and acid-base status in critically ill patients. Misinterpretation of ABG can lead to delayed or inappropriate management of critical conditions such as respiratory failure, metabolic disturbances, and sepsis, increasing morbidity and mortality. ABG interpretation is essential for timely and effective clinical decision-making. Accurate interpretation of ABG results is essential but challenging, especially when both respiratory and metabolic disturbances are present. This study aims to evaluate and enhance the quality of ABG interpretation among healthcare professionals at Aswan University Hospitals, following the American Thoracic Society (ATS) guidelines.

Materials and methods: This prospective cross-sectional study was conducted at Aswan University Hospitals from August to October 2024, involving 273 healthcare professionals, including house officers, medical officers, registrars, and residents. Participants were assessed in two audit cycles: the first without intervention and the second following targeted educational interventions. These interventions, implemented over two weeks, included lectures, educational posters, and focus group discussions focused on ABG interpretation. Data were collected using a validated questionnaire and analyzed to evaluate changes in ABG interpretation accuracy.

Results: The results revealed significant improvements in ABG interpretation skills between the two cycles. The accuracy in verifying ABG results increased from 71.8% to 90.2%, distinguishing acidemia and alkalemia improved from 82.9% to 95.4%, and differentiating respiratory and metabolic disturbances rose from 82.1% to 96.7%. Additionally, proficiency in assessing compensatory mechanisms and calculating the anion gap showed substantial gains, with correct interpretation rates improving from 47% to 78.4% and 69.2% to 98%, respectively.

Conclusion: The educational interventions effectively enhanced ABG interpretation skills among healthcare professionals, demonstrating the importance of targeted training in promoting accurate diagnosis and effective patient care. This study highlights the need for ongoing training in ABG analysis to improve diagnostic accuracy and clinical decision-making in critical care settings.

## Introduction

Arterial blood gas (ABG) analysis is an indispensable diagnostic tool in healthcare, particularly for assessing a patient’s oxygenation, ventilation, and acid-base status. It plays a crucial role in managing critically ill patients, especially those with respiratory conditions or in critical care settings. ABG provides valuable information on ventilation, oxygen delivery, and the body’s acid-base balance, helping healthcare practitioners evaluate interventions such as oxygen therapy or respiratory support. Despite advances in non-invasive monitoring, ABG analysis remains essential, providing life-saving information for accurate diagnosis and treatment [1-5].

Interpreting ABG results, however, can be complex, particularly when both respiratory and metabolic disturbances are present. Acid-base imbalances are common but often misinterpreted, and understanding the relationship between the lungs and kidneys is vital for identifying the dominant disturbance. This understanding helps guide therapeutic decisions in critically ill patients. The transition from identifying disturbances to applying ABG parameters involves bridging theoretical knowledge with clinical acumen, ensuring tailored interventions for each patient scenario. Although many healthcare professionals are familiar with ABG analysis, the varying levels of understanding and the complexity of interpreting results highlight the need for a more thorough grasp of acid-base balance in clinical practice [6-9].

ABG analysis measures key parameters such as pH, partial pressure of carbon dioxide in arterial blood (PaCO_2_), partial pressure of oxygen in arterial blood (PaO_2_), and bicarbonate (HCO_3_-), providing a comprehensive overview of a patient's respiratory and metabolic status. These measurements reflect gas exchange in the lungs and help determine the adequacy of oxygenation and ventilation. Factors such as age and altitude can influence results, requiring individualized interpretation. Maintaining pH within the normal physiological range is critical for numerous cellular processes, and deviations trigger compensatory mechanisms to restore balance, underlining the significance of precise interpretation in clinical care [10].

## Materials and methods

This prospective cross-sectional study was carried out at Aswan University Hospitals in 2024, targeting various medical professionals, including house officers, medical officers, registrars, and residents. The study aimed to evaluate the quality of ABG interpretation in line with the American Thoracic Society (ATS) guidelines [11], identify areas of improvement, and implement strategic interventions to enhance overall performance. The study was conducted from August 1, 2024, to October 31, 2024.

Parameters 

The following are the parameters to be followed: (1) check if the ABG results make sense by using a formula to compare the pH and other values; (2) identify acid-base disturbances; (3) identify whether the issue is respiratory or metabolic by looking at the relationship between pH and PaCO_2_ changes; (4) check if the body is compensating properly for the main problem; (5) if there is metabolic acidosis, calculate the anion gap to see if it is higher than normal; and (6) if the anion gap is higher than normal, see how it relates to the drop in HCO_3_- to determine if another issue might be present.

Audit area/population

The audit was conducted within Aswan University Hospitals, where junior healthcare professionals across various departments were involved in the audit cycles. The focus was on improving the knowledge and accuracy of ABG interpretation by assessing the performance of participants before and after implementing targeted interventions based on ATS guidelines.

Inclusion criteria: (1) junior doctors, including interns and residents, working at Aswan University Hospital and (2) doctors at Aswan University Hospital responsible for interpreting ABG as part of patient care

Exclusion criteria: (1) senior doctors, including consultants and attending physicians at Aswan University Hospital; (2) doctors from outside Aswan University Hospital; and (3) any ABG interpretations conducted outside the designated audit period.

Sample size and sampling technique

A total of 273 participants took part in the study, with 120 individuals included in the first cycle and 153 individuals involved in the second cycle. The sample included house officers, medical officers, registrars, and residents. The sampling method used was simple random sampling, ensuring a representative and balanced selection of participants from both cycles.

Data collection method and analysis

Data collection was conducted using a validated, pre-designed questionnaire, ensuring consistency in responses across both audit cycles. The questionnaire was thoroughly reviewed for completeness by trained doctors before being distributed. All data were analyzed using Google Forms, and improvements in ABG interpretation practices between the two cycles were evaluated.

Audit cycles

The audit was divided into three distinct phases:

First cycle: The first cycle of the audit was conducted from August 1 to August 31, 2024. During this phase, baseline data were collected without any interventions. The assessment focused on participants' knowledge and practice regarding ABG interpretation, utilizing clinical scenario questions derived from the American Thoracic Society's (ATS) standards and guidelines. These questions were designed to assess the participants' understanding of key concepts such as the interpretation of acid-base balance, identification of respiratory and metabolic disturbances, and the management of patients based on ABG results.

Intervention period: The intervention period took place from September 1 to September 15, 2024. Targeted interventions were introduced to improve the quality of ABG interpretation. These interventions included educational posters distributed in internal medicine, pulmonary wards, and hospital reception; lectures conducted by senior doctors; and focus group discussions that covered key topics related to the ATS guidelines. The objective was to enhance participants' knowledge and skills regarding ABG interpretation.

Second cycle: The second cycle was conducted from September 16 to October 31, 2024, following the implementation of the interventions. The same group of participants was reassessed during this phase, using clinical scenario questions that maintained the same conceptual framework as in the first cycle but included modifications to prevent recall bias. These modified questions continued to focus on critical concepts related to acid-base disturbances and management but were altered in wording and context to ensure a fresh assessment of understanding. This ensured that the results accurately reflected any improvements in understanding and application of the ATS guidelines as a result of the interventions.

Ethical considerations

The audit received formal approval from the Aswan University Hospitals Institutional Review Committee under audit number 1003/10/24. All participants provided consent to participate.

## Results

First cycle results

During the initial cycle, the audit revealed varying levels of accuracy among house officers across six key parameters of ABG interpretation. Specifically, 71.8% of participants correctly verified ABG results by assessing coherence between pH, PaCO_2_, and HCO_3_- levels. In terms of identifying acidemia or alkalemia, 82.9% accurately determined the primary acid-base disturbance, while 82.1% were able to differentiate respiratory from metabolic disturbances. However, only 47% could accurately assess the body's compensatory response, and 69.2% calculated the anion gap correctly. Furthermore, 64.1% demonstrated the ability to link an elevated anion gap to changes in HCO_3_-, essential for diagnosing additional acid-base disorders.

Second cycle results (post-intervention)

Following targeted educational interventions, improvements were observed in all six parameters. Accuracy in verifying ABG results rose to 90.2%, while the ability to identify acidemia or alkalemia improved to 95.4%. Participants’ accuracy in distinguishing respiratory versus metabolic disturbances increased to 96.7%, and the correct interpretation of compensatory mechanisms rose significantly to 78.4%. The proportion of participants accurately calculating the anion gap reached 98%, and 89.5% effectively linked an elevated anion gap with HCO_3_- changes, demonstrating enhanced competency in handling complex acid-base cases. These improvements highlight the positive impact of structured training on ABG interpretation skills among junior doctors.

**Table 1:** Records from the first and second cycles, showing the respective percentage of improvement.

Standards	First cycle, N =120	Second cycle, N =153	Percentage of improvement
Check if the ABG results make sense by using a formula to compare the pH and other values	71.8%	90.2%	25.6%
Identify acid-base disturbances	82.9%	95.4%	15%
Identify whether the issue is respiratory or metabolic by looking at the relationship between pH and PaCO_2_ changes	82.1%	96.7%	17.7%
Check if the body is compensating properly for the main problem	47%	78.4%	66.8%
If there's metabolic acidosis, calculate the anion gap to see if it's higher than normal	69.2%	98%	41.6%
If the anion gap is higher than normal, see how it relates to the drop in bicarbonate (HCO_3_-) to determine if another issue might be present	64.1%	89.5%	39.6%

## Discussion

This clinical audit aimed to evaluate the accuracy and quality of ABG interpretation among junior doctors at Aswan Teaching Hospital. The ability to correctly interpret ABG results is crucial for the diagnosis and management of various medical conditions, including acid-base disturbances, respiratory failure, and metabolic abnormalities. The findings from this audit suggest that, while junior doctors demonstrate a basic understanding of ABG interpretation, there are significant gaps in their ability to handle more complex cases that require advanced interpretation skills.

In terms of performance, junior doctors were generally able to interpret straightforward ABG results with reasonable accuracy. However, when faced with more complicated scenarios, errors in interpretation were more prevalent. These types of errors are particularly concerning in critical care settings, where the timely and correct interpretation of ABG results is essential for making quick clinical decisions. Inappropriate interpretation or misdiagnosis could delay treatment, leading to potential harm to patients.

The audit further revealed a clear disparity between theoretical knowledge and practical application of ABG interpretation. Although ABG interpretation is a part of the curriculum during medical school and junior doctor training, many junior doctors struggle to apply this knowledge in clinical situations. This gap suggests that the training currently provided to junior doctors is insufficient in preparing them to handle the complexities of ABG interpretation. This issue is likely exacerbated by the fact that ABG interpretation is often considered a routine task, and junior doctors may not receive adequate supervision or feedback when dealing with more difficult cases.

In response to these findings, it is evident that there is a need for targeted educational interventions. Incorporating more focused training on ABG interpretation into the induction and ongoing professional development programs for junior doctors could improve their competency. This training should not only cover the basic concepts but also provide ample opportunities for junior doctors to practice interpreting ABG results in a variety of clinical scenarios, including more complex cases. Case-based learning, simulation exercises, and mentorship programs would be beneficial to help junior doctors gain confidence in their skills and improve their clinical decision-making.

Another significant aspect highlighted by this audit is the absence of previous clinical audits or studies examining ABG interpretation, both locally and internationally. This lack of research is surprising, considering the importance of ABG interpretation in clinical practice. The scarcity of studies in this area suggests that ABG interpretation may not have been systematically evaluated as a skill that requires ongoing assessment and improvement. The lack of local and international data also points to the need for further research to explore the challenges and gaps in ABG interpretation among healthcare providers, particularly in junior doctor populations.

Given the findings of this audit, it is clear that there is a significant gap in both training and assessment in this area. The lack of established standards or benchmarks for ABG interpretation among junior doctors further complicates efforts to address these deficiencies. It is essential that future audits focus on assessing ABG interpretation skills across different institutions and countries, which could help identify common challenges and inform the development of standardized training programs.

Limitations 

The sample size of 273 participants, though adequate for initial insights, limits the generalizability of the findings, as a larger sample could reveal additional factors influencing ABG interpretation. Furthermore, as a single-center study conducted exclusively at Aswan University Hospital, the results may not fully apply to other settings with differing resources or training structures. Additionally, the brief intervention period may have constrained the depth of knowledge acquired by participants, potentially impacting the retention and long-term application of these skills.

## Conclusions

This audit achieved significant improvements in the ABG interpretation skills of junior doctors at Aswan University Hospital by implementing targeted educational interventions. Specifically, it enhanced their ability to identify acid-base disturbances, assess compensatory mechanisms, and accurately calculate the anion gap, which are essential skills for managing critically ill patients. The audit also highlighted areas where house officers initially struggled, helping tailor future training. Ultimately, the findings demonstrate that structured, targeted education can bridge knowledge gaps, foster diagnostic accuracy, and support safer clinical decision-making. The audit underscores the importance of regular skill assessment and continued education to maintain and improve ABG interpretation skills, ensuring high standards of patient care and promoting efficient clinical practice.

## Appendices

First cycle questionnaire 

Scenario 1

A 45-year-old female patient with a history of chronic obstructive pulmonary disease (COPD) is admitted to the hospital for worsening shortness of breath. The doctor orders an ABG test to evaluate her respiratory status.

ABG results:

- pH: 7.30

-PaCO_2_: 60 mmHg

- HCO_3_-: 28 mEq/L

-PaO_2_: 70 mmHg

Options:

Do these ABG results make sense?

   - A. Yes, the results are consistent with chronic respiratory acidosis with partial metabolic compensation.

   - B. No, the pH should be more acidic given the PaCO_2_ level.

   - C. No, the HCO_3_- level should be lower to match the pH value.

   - D. Yes, the results suggest metabolic acidosis with respiratory compensation.

Scenario 2

A 60-year-old male with a history of congestive heart failure presents with confusion and lethargy. An ABG is performed to assess his condition.

ABG results:

- pH: 7.50

- PaCO_2_: 30 mmHg

- HCO_3_-: 24 mEq/L

- PaO_2_: 85 mmHg

Options:

Based on the pH value, what is the patient's acid-base status?

   - A. Acidemia

    - B. Alkalemia

    - C. Normal pH

    - D. Compensated acidosis

Scenario 3

A 35-year-old woman was brought to the emergency department after experiencing a panic attack, during which she was hyperventilating. The attending physician orders an ABG to assess her condition.

ABG results:

- pH: 7.55

- PaCO_2_: 25 mmHg

- HCO_3_-: 22 mEq/L

 - PaO_2_: 98 mmHg

Options:

What is the primary issue based on the relationship between pH and PaCO^2^?

   - A. Metabolic acidosis.

   - B. Respiratory alkalosis

   - C. Metabolic alkalosis.

   - D. Respiratory acidosis

Scenario 4

A 28-year-old male with a history of diabetic ketoacidosis (DKA) is admitted with nausea, vomiting, and abdominal pain. An ABG is performed.

ABG results:

- pH: 7.20

- PaCO_2_: 25 mmHg

- HCO_3_-: 10 mEq/L

- PaO_2_: 90 mmHg

Options:

Is the body compensating adequately for the primary issue?

   - A. Yes, the PaCO_2_ is appropriately decreased, indicating compensation.

   - B. No, the PaCO_2_ should be higher to indicate compensation.

   - C. No, the HCO_3_- should be increased to indicate compensation.

   - D. Yes, the HCO_3_- is appropriately decreased, indicating compensation.

Scenario 5

A 50-year-old male with a history of alcoholism presents to the emergency department with confusion and abdominal pain. The physician orders an ABG and a basic metabolic panel.

ABG results:

- pH: 7.25

- PaCO_2_: 28 mmHg

- HCO_3_-: 14 mEq/L

- PaO_2_: 88 mmHg

Serum electrolytes:

- Na+: 138 mEq/L.

- Cl-: 100 mEq/L

Options:

What is the anion gap, and what does it suggest?

   - A. Anion gap is 12 mEq/L, which is normal, indicating no additional issues.

   - B. Anion gap is 24 mEq/L, elevated, suggesting a possible cause like lactic acidosis or ketoacidosis.

   - C. Anion gap is 8 mEq/L, which is low, indicating non-anion gap acidosis.

   - D. Anion gap is 16 mEq/L, suggesting metabolic alkalosis.

Scenario 6

A 65-year-old woman with a history of renal failure presents with severe vomiting and altered mental status. An ABG is ordered along with serum electrolytes.

ABG results:

- pH: 7.30

- PaCO_2_: 35 mmHg

- HCO_3_-: 18 mEq/L

- PaO_2_: 92 mmHg

Serum electrolytes:

- Na+: 142 mEq/L.

- Cl-: 98 mEq/L.

- Anion Gap: 26 mEq/L

Options:

Given the elevated anion gap, what does the relationship between the anion gap and the drop in HCO_3_- suggest?

- A. No other underlying issue; the anion gap and HCO_3_- levels are unrelated.

- B. Another metabolic disorder might be present, such as lactic acidosis or ketoacidosis.

- C. The elevated anion gap is solely due to respiratory compensation.

- D. The relationship suggests metabolic alkalosis.

Second cycle questionnaire 

Scenario 1: A 55-year-old male with a history of obesity hypoventilation syndrome is admitted to the hospital with worsening shortness of breath. The doctor orders an ABG test to evaluate his respiratory status.

ABG results:

pH: 7.30

PaCO_2_: 60 mmHg

HCO_3_-: 28 mEq/L

PaO_2_: 70 mmHg

Options:

Do these ABG results make sense?

A. Yes, the results are consistent with chronic respiratory acidosis with partial metabolic compensation.

B. No, the pH should be more acidic given the PaCO_2_ level.

C. No, the HCO_3_- level should be lower to match the pH value.

D. Yes, the results suggest metabolic acidosis with respiratory compensation.

Correct Answer: A

Scenario 2: A 65-year-old female with a history of chronic renal insufficiency presents with confusion and lethargy. An ABG is performed to assess her condition.

ABG results:

pH: 7.50

PaCO_2_: 30 mmHg

HCO_3_-: 24 mEq/L

PaO_2_: 85 mmHg

Options:

Based on the pH value, what is the patient's acid-base status?

A. Acidemia

B. Alkalemia

C. Normal pH

D. Compensated acidosis

Correct Answer: B

Scenario 3: A 40-year-old man is brought to the emergency department after experiencing a panic attack, during which he was hyperventilating. The attending physician ordered an ABG to assess his condition.

ABG results:

pH: 7.55

PaCO_2_: 25 mmHg

HCO_3_-: 22 mEq/L

PaO_2_: 98 mmHg

Options:

What is the primary issue based on the relationship between pH and PaCO_2_?

A. Metabolic acidosis

B. Respiratory alkalosis

C. Metabolic alkalosis

D. Respiratory acidosis

Correct Answer: B

Scenario 4: A 32-year-old female with a history of acute kidney injury is admitted with nausea, vomiting, and abdominal pain. An ABG is performed.

ABG results:

pH: 7.20

PaCO_2_: 25 mmHg

HCO_3_-: 10 mEq/L

PaO_2_: 90 mmHg

Options:

Is the body compensating adequately for the primary issue?

A. Yes, the PaCO_2_ is appropriately decreased, indicating compensation.

B. No, the PaCO_2_ should be higher to indicate compensation.

C. No, the HCO_3_- should be increased to indicate compensation.

D. Yes, the HCO_3_- is appropriately decreased, indicating compensation.

Correct Answer: A

Scenario 5: A 45-year-old male with a history of severe diarrhea presents to the emergency department with confusion and abdominal pain. The physician orders an ABG and a basic metabolic panel.

ABG results:

pH: 7.25

PaCO_2_: 28 mmHg

HCO_3_-: 14 mEq/L

PaO_2_: 88 mmHg

Serum electrolytes:

Na+: 138 mEq/L

Cl-: 100 mEq/L

Options:

What is the anion gap, and what does it suggest?

A. Anion gap is 12 mEq/L, which is normal, indicating no additional issues.

B. Anion gap is 24 mEq/L, elevated, suggesting a possible cause like lactic acidosis or ketoacidosis.

C. Anion gap is 8 mEq/L, which is low, indicating non-anion gap acidosis.

D. Anion gap is 16 mEq/L, suggesting metabolic alkalosis.

Correct Answer: B

Scenario 6: A 70-year-old male with a history of chronic respiratory failure presents with severe vomiting and altered mental status. An ABG is ordered along with serum electrolytes.

ABG Results:

pH: 7.30

PaCO_2_: 35 mmHg

HCO_3_-: 18 mEq/L

PaO_2_: 92 mmHg

Serum electrolytes:

Na+: 142 mEq/L

Cl-: 98 mEq/L

Anion Gap: 26 mEq/L

Options:

Given the elevated anion gap, what does the relationship between the anion gap and the drop in HCO_3_- suggest?

A. No other underlying issue; the anion gap and HCO_3_- levels are unrelated.

B. Another metabolic disorder might be present, such as lactic acidosis or ketoacidosis.

C. The elevated anion gap is solely due to respiratory compensation.

D. The relationship suggests metabolic alkalosis.

Correct Answer: B
